# The role of natural killer cells in liver inflammation

**DOI:** 10.1007/s00281-021-00877-6

**Published:** 2021-07-07

**Authors:** A. J. Highton, I. S. Schuster, M. A. Degli-Esposti, M. Altfeld

**Affiliations:** 1 Heinrich Pette Institute, Leibniz Institute for Experimental Virology, Hamburg, Germany; 2 Experimental and Viral Immunology, Department of Microbiology and Infection and Immunity Program, Monash Biomedicine Discovery Institute, Monash University, Clayton, Victoria Australia; 3 Experimental Immunology, Lions Eye Institute, Perth, Western Australia Australia; 4 Institute for Immunology, University Medical Centre Hamburg-Eppendorf, Hamburg, Germany

**Keywords:** Liver inflammation, NK cells, Viral hepatitis, Autoimmune liver disease, Fatty liver disease

## Abstract

The liver is an important immunological site that can promote immune tolerance or activation. Natural killer (NK) cells are a major immune subset within the liver, and therefore understanding their role in liver homeostasis and inflammation is crucial. Due to their cytotoxic function, NK cells are important in the immune response against hepatotropic viral infections but are also involved in the inflammatory processes of autoimmune liver diseases and fatty liver disease. Whether NK cells primarily promote pro-inflammatory or tolerogenic responses is not known for many liver diseases. Understanding the involvement of NK cells in liver inflammation will be crucial in effective treatment and future immunotherapeutic targeting of NK cells in these disease settings. Here, we explore the role that NK cells play in inflammation of the liver in the context of viral infection, autoimmunity and fatty liver disease.

## Introduction

The liver is a central organ involved in digestion, metabolism and detoxification of blood. The majority of blood entering the liver comes via the hepatic portal vein from the spleen and gastrointestinal tract. This venous blood carries antigens, which are subsequently exposed to the liver immune repertoire, positioning the liver as an important immunological site for both immune tolerance and activation. Dysregulation of these immunological processes can affect both liver and systemic immune responses. The liver is home to a variety of immune cells including Kupffer cells, MAIT cells, γδ T cells, αβ T cells, B cells, NKT cells, ILCs and NK cells [1]. Among these, NK cells make up 40% of total lymphocytes in the liver of humans [2], indicating the potential for an important role for NK cells in regulating liver immunity. NK cells are important in early innate immune responses but can also regulate other innate and adaptive immune responses [3] and have been suggested to play an important role in the pathogenesis of several liver diseases, including autoimmune diseases of the liver and hepatotropic viral infections [4]. The involvement of NK cells in hepatocellular carcinoma (HCC) (reviewed in [5, 6]), though important, is beyond the scope of this review and not discussed.

**Fig. 1:**
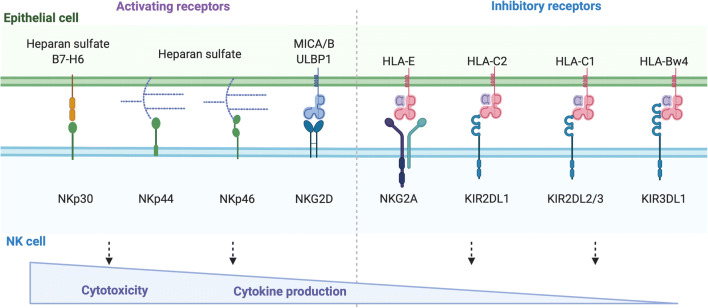
Activating and inhibitory receptors expressed on NK cells. NK cell-activating receptors binding to their respective ligands promote activation and subsequent cytotoxicity and cytokine production, while inhibitory receptors oppose this response. *Created with* BioRender.com

NK cells can both lyse infected or cancerous cells and also produce pro-inflammatory cytokines such as interferon gamma (IFNγ). The function of NK cells is tightly regulated via signalling through both activating and inhibitory receptors expressed on their surface. In humans, inhibitory killer Ig-like receptors (KIRs) primarily bind to human leukocyte antigen (HLA)-B and HLA-C molecules, while the inhibitory receptor NKG2A binds to HLA-E [7–10] (Fig. 1). The interactions with these inhibitory NK cell receptors ensure that healthy cells expressing HLA class I molecules are not inappropriately targeted by NK cells. Activating receptors such as NKp30, NKp44, NKp46 and NKG2D and activating KIRs are also expressed by NK cells and promote NK cell activation upon binding to their ligands, which include stress molecules and molecules upregulated in response to inflammation [11]. A combination of loss of binding of inhibitory receptors and engagement of activating receptors results in NK cell activation and can facilitate killing of virally infected cells, cancerous cells or inappropriately activated cells.

**Fig. 2:**
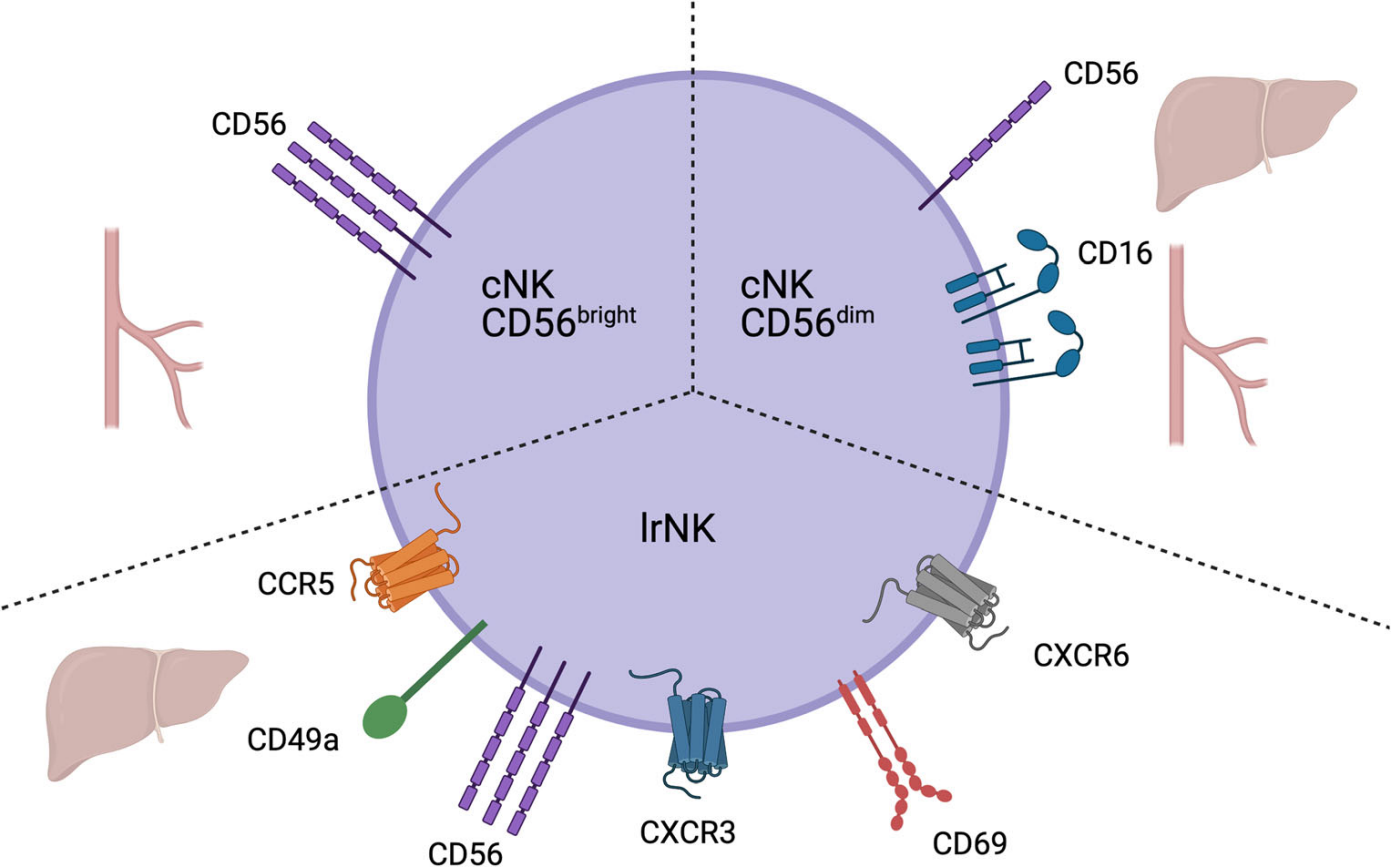
Subsets of NK cells present within the liver and vasculature. CD56^bright^ cNK cells are located primarily within the vasculature. CD56^dim^ cNK cells can be found within the vasculature and also in the liver parenchyma. Resident within the liver are lrNK cells expressing CCR5, CXCR3, CXCR6, CD69, CD49a and CD56. *Created with* BioRender.com

Tissue-resident populations of NK cells have been described in multiple organs with distinct phenotype and function, indicating specific interactions between these tissues and NK cells [12]. Indeed, the liver possesses a distinct population of liver-resident NK cells (lrNKs) important for the immune function of this organ, along with conventional NK cells (cNKs) (Fig. 2). While cNKs circulate through the liver and vasculature, lrNKs are believed to remain in the liver [13]. cNKs are broadly categorised based on expression of the adhesion molecule CD56 as CD56^bright^ or CD56^dim^ NK cells, with the latter subset being the most numerous in peripheral blood [14]. In contrast, CD56^bright^ NK cells are largely enriched in the liver compared to the blood, with almost equal frequencies of CD56^bright^ compared to CD56^dim^ NK cells [15]. These CD56^bright^ lrNKs can be further defined based on their expression of CD69, CD49a, CCR5 and CXCR6 [13]. CD69 suppresses tissue egress through association with sphingosine-1-phosphate receptor 1, and CD49a is a collagen-binding integrin, thus promoting tissue retention [16, 17]. The presence of CCL3, CCL5 and CXCL16 in liver sinusoids further promotes the retention of NK cells expressing the cognate receptors CCR5 and CXCR6 [13]. lrNKs have been shown to be not only phenotypically different from their CD56^bright^ cNK brethren but also transcriptionally distinct [18], and as with other tissues [19], these resident NK cells appear to be specifically suited to function in the liver environment and integral to the regulation of immune function in this organ. Here, we discuss liver NK cells and their involvement in the pathogenesis of liver inflammation and diseases following dysregulation.

## NK cells in the liver

**Fig. 3:**
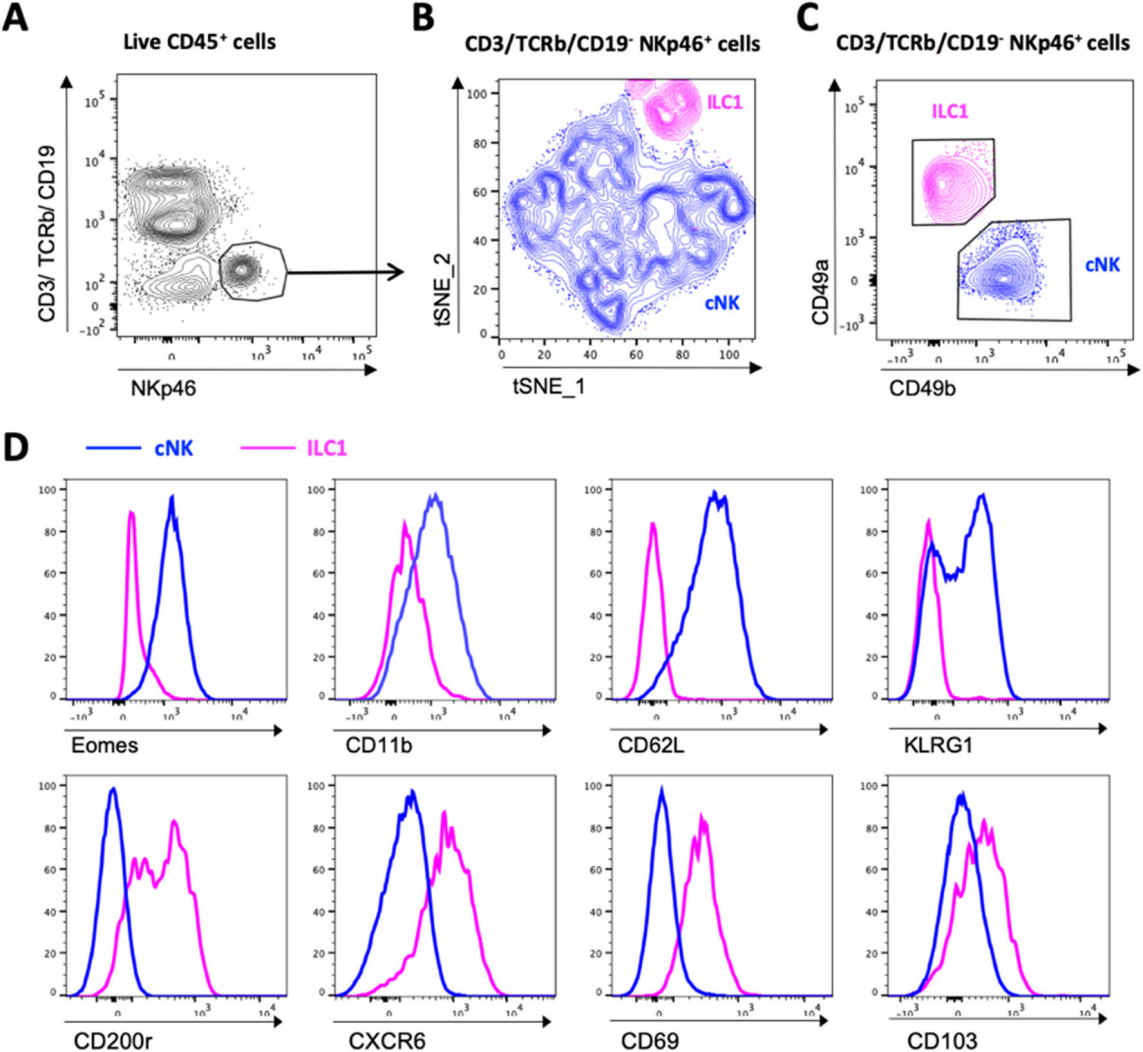
Distinct phenotypes characterise mouse liver cNK cells and ILC1s at steady state. Liver leukocytes from naïve BALB/c mice were prepared for flow cytometric analysis. **A** Single live CD45^+^ cells were gated for CD3^-^ TCRβ^-^ CD19^-^ NKp46^+^ cells, and **B** unsupervised nonlinear dimensional reduction using t-distributed stochastic neighbourhood embedding (tSNE) analysis was performed. Distinct clustering of cNK cells (blue population) and ILC1s (pink population) is shown. **C** Total NKp46^+^ cells analysed for CD49a and CD49b expression to distinguish ILC1s and cNK cells. **D** Histogram overlays for the indicated marker expression in ILC1s and cNK cells are shown. Data are concatenated from 3 mice and are representative of 3 independent experiments

Liver-resident NK-like cells (NK1.1^+^ CD3^-^) were first described in the mouse and were initially distinguished from cNK cells by the absence of the mature NK cell marker CD49b and high TRAIL expression at steady state [20]. The identification of an increasing number of tissue-resident NK-like cells, not only in the liver but also in other non-lymphoid tissues, led to the introduction of a new nomenclature, which classifies most tissue-resident NK-like cells as innate lymphoid cells type 1 (ILC1) in the mouse (reviewed in Vivier et al. 2018 [21]). Although ILC1s have also been described in humans, NK cells and ILC1s are difficult to distinguish due to the lack of specific markers. For the purpose of this review, we will therefore refer to human liver-resident NK cells and ILC1s as lrNK cells. The transcription factors required for mouse liver ILC1 cell development are distinct from their cNK counterparts: murine ILC1s depend on T-bet and Hobit, while cNK cells require Eomes for their development [22–24]. Liver ILC1s have recently been reported to develop locally, with IFNγ production by liver ILC1s promoting their development from IFNγR^+^ liver progenitors [25]. Transfer and parabiosis studies have confirmed that hepatic ILC1s home back to the liver where they are maintained without recirculating, thus identifying them as *bona fide* tissue-resident cells [26, 27]. Further investigations revealed extensive phenotypic differences between ILC1 and cNK populations [21, 26, 27]. Figure 3 provides an overview of murine liver cNK cells and ILC1s at steady state. Unsupervised dimensional reduction analysis of NKp46^+^ cells in the liver (Fig. 3A) shows distinct clustering of ILC1s and cNK cells (Fig. 3B). ILC1s are distinguished as CD49a^+^ CD49b^-^ cells (Fig. 3C), which lack the expression of Eomes, CD11b, CD62L and KLRG1 but express CD200R, CXCR6, CD69 and CD103, while cNK cells present with the reverse phenotype at steady state (Fig. 3D). Furthermore, unlike cNK cells, ILC1s have limited cytotoxic potential but are capable of mounting a strong cytokine response [21].

Subsequent to the characterisation in the mouse, human CD56^bright^ CD16^-^ lrNK cells were distinguished from hepatic cNK cells based on the expression of CD69 and the chemokine receptors CXCR6 and CCR5 [13, 18]. Expression of these receptors specifically occurs on lrNK cells, but not on cNK cells, and the chemokine receptors CCR5 and CXCR6 are thought to mediate localisation to liver sinusoids, a compartment rich in the expression of the respective ligands CCL3, CCL5 and CXCL16 [13]. CXCR6^+^ lrNK cells express Eomes but lack T-bet along with a number of KIR molecules [13, 18]. Functionally, CXCR6^+^ lrNK cells produce less IFNγ, tumour necrosis factor (TNF) and macrophage inflammatory protein (MIP)-1β in response to stimulation than hepatic cNK cells. They also express less perforin and Granzyme B but show enhanced Granzyme K expression and are capable of degranulation [28]. Finally, a study investigating hepatic NK cells in liver transplants ascertained that, although CXCR6^+^ lrNK cells are long-lived and do not recirculate, they could still be replenished from the circulation [28]. In addition to CXCR6^+^ CD56^bright^ CD16^-^ lrNK cells, a second liver-resident NK cell population has been described [29]. These cells are CD56^bright^ CD16^-^, lack CXCR6 expression and instead are identified by expression of CD49a—similar to mouse ILC1s [26, 29]. CD49a^+^ lrNK were present in approximately 40% of tested individuals with a frequency of approximately 2.3% of hepatic CD56^bright^ cells. CD56^bright^ CD49a^+^ NK cells specifically localised to the liver parenchyma and, unlike CXCR6^+^ lrNKs, expressed T-bet but not Eomes. They also exhibited a pattern of KIR and NKG2C expression indicative of clonal-like expansion, although they did not express CD57, a marker expressed on differentiated cNK cells. Furthermore, CD56^bright^ CD49a^+^ lrNK cells had reduced capacity to degranulate compared to hepatic CD49a^-^ NK cells but were potent producers of cytokines such as IFNγ, TNF and GM-CSF.

Overall, there is compelling evidence to support that the liver harbours multiple distinct subsets of resident as well as circulating NK cells at steady state. We are only beginning to understand the roles that these NK cell subsets play in response to immunological challenges, with many questions still unanswered, such as their roles in maintaining liver homeostasis or during hepatic insults. In this regard, the phenotype, function and anatomical localisation of lrNK and hepatic cNK may provide insights about their specific roles in maintaining overall liver health. For instance, it is highly plausible that sinusoidal lrNK cells may play a role in shaping the responsiveness of other sinusoidal cells, such as Kupffer cells. Interestingly, it has been reported that murine liver ILC1s can generate antigen-specific recall responses in a model of contact hypersensitivity [26, 30]. By extension, human CD49a^+^ lrNK cells may therefore represent an analogous memory-like population, as they are characterised by the expression of a receptor repertoire consistent with clonal expansion [29]. However, how tissue-resident cells can induce antigen-specific responses in distal sites remains to be understood. Below, we review the roles of hepatic NK cell populations in liver homeostasis and in the context of different inflammatory settings.

## NK cells in liver homeostasis and tolerance

The liver is continuously exposed to microorganisms, microbial products and food antigens delivered through the portal vein from the intestine. This necessitates an increased level of immune tolerance to avoid excessive inflammation, tissue damage and loss of liver function. This immunological status is mediated by a complex interplay of hepatic cells and immune cells, including NK cells. NK cells can shape, and in turn are shaped, by the hepatic microenvironment via both direct cell-to-cell contact and the production of soluble factors. Kupffer cells are liver sinusoid-resident macrophages that play an important role in activating NK cells via the expression of interleukin (IL)-18 following the detection of microbial products [31]. Simultaneously, Kupffer cells can also suppress NK cell activity through the production of IL-10 that leads to inhibition of IFNγ expression and renders NK cells hyporesponsive [31, 32]. In addition, the exposure of hepatic NK cells to apoptotic cells has been shown to induce transforming growth factor beta (TGF-β) expression by NK cells, which acts in an autocrine manner to reduce NK cell IFNγ production [33]. The roles of hepatic lrNK and cNK cell populations in regulating liver homeostasis and tolerance are not well delineated and thus require further investigation to dissect their specific contributions. Transcriptional profile analyses of murine hepatic NK cells and ILC1s revealed increased expression of genes associated with immune regulation in resident ILC1s and an increase in genes associated with cytotoxic function in cNK cells [34]. Consistent with this, hepatic ILC1s are capable of providing protection against acute liver injury [35]. Following the initiation of liver damage, murine ILC1s are activated via IL-12 to produce IFNγ, which induces the upregulation of the antiapoptotic factor Bcl-xL in hepatocytes. These findings stand in contrast to previous reports linking NK cell IFNγ production to increased liver damage and impaired regeneration in models of partial hepatectomy with concomitant viral infection or toll-like receptor (TLR) 3 ligand administration [36, 37]. It is important to note that these studies did not dissect the respective roles of lrNK and cNK cells. A possible explanation for the disparate findings on the role of NK cell IFNγ production may thus be due to cytokine release by cNK cells in distinct niches within the liver, as well as concentration dependent effects of IFNγ.

## NK cells in a diseased liver

**Fig. 4:**
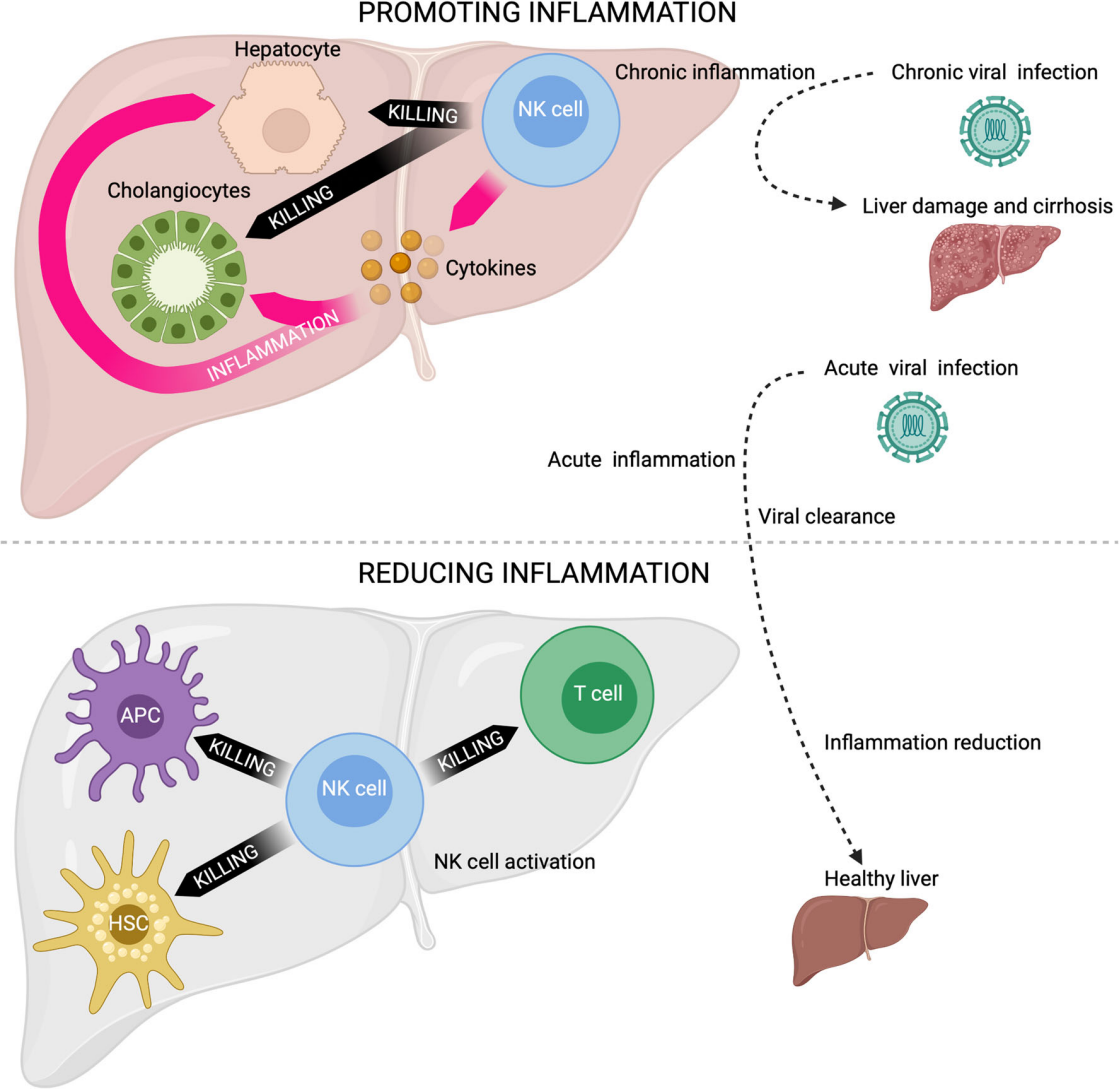
Potential role of NK cells in promoting and reducing liver inflammation. NK cells can promote liver inflammation through the secretion of pro-inflammatory cytokines and the killing of hepatocytes and cholangiocytes. On the other hand, NK cells can reduce liver inflammation through the killing of activated T cells and other liver-resident cells. *Created with* BioRender.com

NK cells can play a role in multiple liver diseases, including autoimmune diseases of the liver, cancer, fatty liver disease and viral diseases. The impact of NK cells on liver diseases is mediated through their direct cytotoxic function against hepatocytes, cholangiocytes or other immune cells in the liver, including T cells and antigen-presenting cells, and the secretion of cytokines, including IFNγ (Fig. 4). NK cells can thereby either promote or reduce liver inflammation. Under normal circumstances, cytotoxic and inflammatory functions of NK cells aid in the clearance of acute disease, thus facilitating a return to homeostasis. However, persistent insults without resolution can lead to chronic liver inflammation due to dysregulation of inflammatory processes. Persistent inflammation can lead to liver fibrosis, which can ultimately progress to cirrhosis and permanent liver damage. Although acute inflammation can be helpful in restoring health, chronic inflammation is a common hallmark of multiple liver diseases facilitated by NK cells, along with other immune cells. The role of cNK cells in the aetiology of a number of liver diseases is discussed below.

## NK cells in autoimmune liver diseases

Dysregulation of the liver’s immune milieu due to both genetic and environmental factors can lead to autoimmune targeting of liver-specific compartments. Three main autoimmune diseases have been described in the liver: primary biliary cholangitis (PBC), primary sclerosing cholangitis (PSC) and autoimmune hepatitis (AIH) [38].

PBC is most prevalent in women and is typically characterised by destruction of the small intrahepatic bile ducts and production of anti-mitochondrial antibodies [40]. Both circulating and liver NK cell frequencies are increased in PBC patients, as is the cytotoxic function of NK cells [39, 40], suggesting that NK cells may be involved in PBC pathogenesis. Indeed, biliary epithelial cells (BECs) have been shown to be destroyed by autologous NK cells at a high NK:BEC ratio [41]. Interestingly, at a low NK:BEC ratio, NK cells protect BECs from subsequent destruction by NK cells via IFNγ-mediated upregulation of HLA, though this renders the BECs susceptible to killing by autoreactive T cells [41], again suggesting that NK cells can promote or reduce disease progression depending on the specific circumstances. Circulating NK cells expressing CXCR6 and CD49a are found in higher frequencies in PBC patients compared to healthy controls, and low-dose IL-12 stimulation has been shown to preferentially increase expression of these markers [42], potentially linking monocyte activation to NK cell dysregulation. Together, these data suggest a propensity for liver homing of NK cells in PBC and a potential role for NK cells in lysing BECs.

PSC occurs equally in both males and females but is more often subclinical in females [43, 44]. PSC is characterised by destruction of both intra- and extra-hepatic bile ducts leading to fibrosis and diffuse inflammation [38]. The frequencies of HLA-Bw4 and HLA-C2, ligands for the inhibitory NK cell receptors KIR3DL1 and KIR2DL1, respectively, were reduced in PSC patients compared to healthy controls, indicating a potential role for NK cells in the disease [45]. PSC patients with less fibrosis were shown to have more hepatic NK cells [46], potentially due to the reported anti-fibrotic role of NK cells in the liver via killing of pro-fibrogenic hepatic stellate cells (HSCs) [47–50]. Increased CCR7 expression has been reported on circulating and intrahepatic NK cells in PSC patients [51], and plasma levels and intrahepatic expression of CCL21, the ligand for CCR7, were increased in PSC patients [51]. Moreover, CCL21 can be produced by CD11c^+^ cells present in the portal tracts of PSC livers [52]. Overall, these data suggest that the recruitment of CCR7-expressing NK cells may be contributing to liver inflammation in PSC patients.

AIH is a chronic liver disease that can present as either type I or type II disease [53]. These two disease types can be distinguished based on the presence of circulating autoantibodies. Anti-nuclear antibodies or anti-smooth muscle antibodies define type I disease, and anti-liver kidney microsomal type-1 or anti-liver cytosol type-1 autoantibodies define type II disease [54–57]. Type I is the most common form of the disease and occurs predominantly in females, while type II is more frequent in girls between the ages of 2 and 14 [56, 58]. In a Japanese cohort, KIR3DL1 and the cognate ligand HLA-B Bw4-80Ile were associated with AIH, while KIR3DL1/HLA-B Bw4-80Thr and KIR2DL1/HLC-C2 were associated with protection from the disease [59]. In a separate study, KIR2DS1 and HLA-C2 were associated with AIH [60]. These associations between KIR/HLA compound genotypes and AIH suggest a potential role for KIR^+^ NK cells in AIH pathogenesis. Furthermore, a recent study showed that a SNP in the HLA-DP beta chain is associated with the risk of developing AIH [61]. Interestingly, this susceptibility SNP tracks a subset of HLA-DP molecules that serve as ligands for the activating NK cell receptor NKp44 [62], further supporting a role of NK cells in AIH pathogenesis. PolyI:C treatment in a mouse model of hepatitis demonstrated recruitment and activation of NK cells in the liver with partial disease abrogation following NK cell depletion [63]. The frequency of circulating CD56^bright^ NK cells was found to be increased in untreated AIH patients prior to corticosteroid treatment [64], whereas decreased frequencies of circulating CD56^dim^ NK cells have been observed in patients with active AIH or while in remission [65]. Interestingly, in a mouse model of AIH, CXCR3^+^ NK cells, similar to human CD56^dim^ NK cells, were enriched in the liver, though both circulating and hepatic NK cells had increased cytotoxic phenotypes [65]. These data further suggest an important role for NK cells in AIH, including possible recruitment of destructive NK cells from the periphery. However, lrNK cells might also modulate the function of antigen-presenting cells or T cells in the liver and thereby reduce liver inflammation in AIH (Fig. 4). Overall, further research is required to understand the precise role of lrNK cells, and particular subpopulations of lrNK cells, in the pathogenesis of autoimmune liver diseases.

## Viral infections of the liver

A number of viruses infect the liver; however, the most prevalent are the hepatotropic hepatitis viruses. There are 5 types of hepatitis viruses, classified as Hepatitis A to E despite these viruses being unrelated to each other [66]. Hepatitis A (HAV) and E (HEV) cause only acute infection, while hepatitis B (HBV), C (HCV) and D (HDV) can establish long-lasting chronic infections. In addition, chronic HBV and HCV infection can lead to the development of life-threatening liver diseases, such as cirrhosis and HCC. Although vaccines are available for HAV, HBV and HDV, and HCV is treatable by direct acting anti-virals (DAA), these viral infections continue to cause considerable morbidity and mortality and carry a significant burden of disease globally [67].

In patients with acute HBV infection, NK cells are highly activated as characterised by elevated expression of activating receptors, reduced expression of inhibitory receptors, increased IFNγ expression and enhanced ability to degranulate compared to NK cells from healthy controls [68, 69]. Importantly, this increased activation state was shown to correlate with the severity of liver damage (likely promoted by the cytolytic capacity of NK cells) as well as improved HBV control (likely via IFNγ production by NK cells) [68]. A more recent study outlined a role for activation of antibody-dependent cellular cytotoxicity (ADCC) by NK cells in responses to acute HBV infection and the generation of both cytotoxic and cytokine responses [70]. In addition to acting as direct effectors, NK cells can promote adaptive responses in a mouse model of acute HBV infection [71]. IFNγ production by hepatic CD49a^-^ CD49b^+^ cNK cells improves anti-viral CD8^+^ T cells responses, leading to enhanced viral clearance [71]. By contrast, during chronic HBV (CHB) infection, NK cells display fewer activating and more inhibitory receptors and lose effector functions, particularly the capacity to secrete cytokines [72, 73]. These findings are consistent with recent studies describing the expansion of dysfunctional CD56^-^ CD16^+^ NK cells and a transcriptional NK cell profile similar to that of exhausted T cells in CHB patients [74, 76]. NK cell dysfunction in CHB patients is mediated by elevated expression of immunosuppressive cytokines such as IL-10 and TGF-β [75, 76]. Blocking these cytokines increased the expression of activating receptors and improved NK cell effector function. Although CHB significantly decreases the functionality of NK cells, numerous studies have reported increased TRAIL expression on both circulating and hepatic NK cells [77–79]. CD56^bright^ TRAIL^+^ NK cells can kill hepatocytes in vitro, and their presence has been associated with the severity of liver damage in patients with CHB-related liver cirrhosis [77, 78]. Furthermore, TRAIL^+^ NK cells have been shown to induce apoptosis of HBV-specific CD8^+^ T cells, which would thereby lead to impaired virus control and increased immunopathology. Thus, TRAIL^+^ NK cells can mediate both liver damage (by killing hepatocytes) and impair anti-viral responses (by killing anti-viral T cells) [79].

Similar to HBV, NK cells are involved in the response to HCV infection. Although the availability of DAAs has significantly improved the outcome of HCV infection, HCV-induced pathologies still pose a major health burden [67], and understanding the role of the immune response to HCV remains highly relevant. Genetic analyses have provided evidence for associations between the expression of specific KIRs and their HLA ligands and the outcome of HVC infection. This is best exemplified by the finding that the KIR2DL3/HLA-C1/C1 genotype is associated with spontaneous resolution of HCV infection [80]. Furthermore, in culture systems, KIR3DS1^+^ NK cells have been shown to recognise and lyse HCV-infected hepatocytes expressing HLA-F and have therefore been postulated to assist in controlling HCV infection [81]. The role of NK cells during acute HCV infection is clear, but the relevant mechanisms remain largely unknown. Although NK cells from infected subjects have been reported to have an enhanced capacity to secrete IFNγ and degranulate compared to those from uninfected controls, these activities do not appear to correlate with the outcome of infection [82]. Interestingly, a recent study has shown that NK cells activated by cytokines present during HCV infection can kill CD4^+^ T cells and has proposed that this may actually promote progression to chronicity [83]. In patients with chronic HCV (CHC) infection, the distribution of NK cell subsets is altered, and there is a relative increase in CD56^bright^ NK cells compared with healthy controls [84, 85]. Furthermore, NK cells display both phenotypic and functional changes. Despite some inconsistencies between different studies, increased expression of activating receptors, such as NKG2D and NKp44, has been reported in NK cells from CHC patients compared to those from healthy controls [73, 86]. A polarisation in NK cell functionality, manifesting as reduced capacity to express IFNγ and TNF but increased degranulation, indicative of greater cytotoxic potential, was noted in CHC patients [86]. This phenotype was attributed to exposure to IFNα as recapitulated by exposing NK cells to this cytokine in vitro [86]. In separate studies, CHC infection was associated with a decrease in NK cell effector function, a phenotype linked to increased expression of the inhibitory receptor CD94/NKG2A [87, 88]. NK cells from CHC patients exhibited higher CD94/NKG2A expression and produced elevated levels of IL-10 and TGF-β when cultured with hepatocytes expressing HLA-E, the CD94/NKG2A cognate ligand [87]. Blocking NKG2A signalling resulted in reduced IL-10 and TGF-β production by NK cells and restored the ability of NK cells to participate in the activation of DC required to prime anti-viral T cell responses [87]. A more recent study, using a humanised mouse model, confirmed that impaired NK cell function contributes to HCV persistence and that blocking NKG2A signalling can revive NK cell IFNγ secretion and improve NK cell cytotoxicity [88]. This increase in IFNγ production led to improved anti-viral CD8^+^ T cell responses and reduced HCV loads. Importantly, chronic HCV infection appears to permanently affect the functionality of NK cells, as the NK cell repertoire remained altered even after successful DAA treatment [89]. How this may affect NK cell responses to other immunological challenges is unknown. The respective roles of lrNK versus circulating NK cells in the response to CHC infection are not well understood. Some insight has been provided by a recent study reporting that CHC patients with lower liver disease scores showed an expansion of hepatic CD56^bright^ CD16^-^ NK cells, a population thought to be resident in the liver [28, 90]. Expansion of CD56^bright^ CD16^-^ lrNK cells also correlated with increased levels of IL-10 in portal vein blood and reduced responsiveness of hepatic lymphocytes to TLR stimulation, compared to circulating lymphocytes [90]. These findings suggest that lrNK cells may be involved in maintaining liver homeostasis in CHC. Overall, it is evident that NK cells play an important role in the immune response to both acute and chronic HBV and HCV infection. Activated NK cells can participate in elimination of virally infected cells and regulation of anti-viral responses, as well as maintenance of liver homeostasis, particularly in the acute stages of infection. Thus, dysregulated NK cell responses are often characterised by diminished effector responses and an inhibitory phenotype allowing for viral persistence and enhance tissue damage. Further studies are required to determine not only which type of NK cell responses are required for optimal viral control at different stages of infection but also the distinct roles that tissue-resident and circulating NK cells play in these processes.

In some instances, viral hepatitis can be caused by members of the *herpesviridae* family, such as cytomegalovirus (CMV) [91]. Although CMV hepatitis is rare in immunocompetent hosts, the virus can cause severe disease in immunocompromised individuals. Acute CMV infection is usually controlled without causing disease, but the virus remains lifelong in a state of latency, and intermittent CMV reactivation occurs but is controlled by the immune system [91]. Immune responses to CMV have been widely studied using mouse models of infection with murine CMV (MCMV). These models have provided extensive insight into the role of NK cells in the immune response to MCMV infection in the liver. Following acute infection, NK cells are recruited into the liver where they contribute to the control of MCMV through cytotoxic and cytokine (mainly IFNγ) responses [92, 93]. During infection, NK cells also play a critical role in regulating macrophage activation in a perforin-dependent manner [94]. In the absence of NK cell-mediated regulation, excessive TNF production by macrophages leads to severe liver damage [94]. During acute MCMV infection, NK cells in the liver also produce IL-10 [95]. Although this cytokine does not contribute to viral control or the initiation of adaptive anti-viral immunity, it limits the magnitude of CD8 T cell responses and consequent pathology in mice lacking perforin [96]. In humans, expansion of CD56^dim^ CD16^+^ NK cells expressing NKG2C has been noted in individuals with active CMV infection [97, 98]; these cells are maintained long term and can expand following exposure to CMV antigen, as noted in allogeneic stem cell transplantation [99]. The role of these NK cells, referred to as “adaptive or memory-like” NK cells, remains largely unknown, and further investigation is required to determine how memory-like NK cells affect CMV infection and reactivation in organs like the liver.

Severe acute respiratory syndrome coronavirus 2 (SARS-CoV-2) is the recently emerged virus at the centre of a global pandemic. SARS-CoV-2 infection primarily targets cells of the respiratory tract but virus has been detected in multiple organs, including the liver [100]. Furthermore, SARS-CoV-2 infection can have a series of extrapulmonary manifestations, including hepatobiliary damage (reviewed in [101]). Acute hepatitis has been reported [102, 103], and increased concentrations of enzymes associated with liver damage (AST, ALT) were found to correlate with the severity of COVID-19 disease [103–105]. COVID-19-associated complications have also been reported in a liver transplant patient, where infection appeared related to the virus being passed by a SARS-CoV-2-positive donor [106]. Enormous efforts have been directed to unravel the complexities of protective versus pathogenic immune responses in the context of SARS-CoV-2 infection. However, current understanding of tissue-specific immunity in tissues such as the liver is still limited. Although tissue inflammation and increased frequencies of immune cells have been observed in livers of COVID patients, the potential contribution of lrNK or cNK cells to SARS-CoV-2 control and/or COVID-19 pathology remains unknown [107]. Analyses of circulating NK cells have revealed a reduction in both CD56^bright^ and CD56^dim^ NK cells in COVID-19 patients compared to healthy controls [108]. The remaining NK cells displayed a more activated phenotype but also appeared to upregulate markers associated with limiting NK cell function (NKG2A), and indeed NK cell effector functions were compromised in severely ill COVID-19 patients [108–110]. Interestingly, an enrichment of “adaptive” NK cells was also observed [108]. A reduction in circulating NK cells has also been reported in children infected by SARS-CoV-2 prompting investigators to suggest that NK cells might have been recruited to sites of infection to provide protective immunity [111]. Overall, NK cells clearly mount a response to SARS-CoV-2 infection, but similar to hepatitis virus infections, they might become dysfunctional during severe disease, and their role in organs like the liver requires further investigation.

In sum, the available evidence demonstrates that during viral infections, populations of NK cells that reside in or are recruited to the liver mediate a plethora of activities. These activities range from those that clearly favour health by providing either anti-viral protective immunity or limiting overt inflammation, to those that are highly pathogenic and contribute to tissue disruption, either directly or by favouring the pathological activities of other cell types. For these reasons, it is imperative to define the role of NK cells in viral infections so that appropriate interventions can be utilised.

## Fatty liver disease

Non-alcoholic fatty liver disease (NAFLD) is the most common chronic liver disease and is characterised by steatosis, the accumulation of lipids, within hepatocytes. Progression of the disease can lead to non-alcoholic steatohepatitis (NASH) involving inflammation and fibrosis. Risk of liver cirrhosis and progression to HCC is increased following transition to NASH. Additionally, NAFLD is often associated with metabolic syndrome along with obesity, high blood pressure and type II diabetes. NAFLD can be controlled early through weight loss and lifestyle changes, but no other treatment is currently available, and the associated co-morbidities complicate treatment if disease progresses further to HCC. Alarmingly, as obesity increases in western nations, so does the frequency of NAFLD and NASH. Evidence from murine models of liver disease have implicated NK cells in disease progression to NASH. In these models, NK cell ligand expression is increased in the liver, thus promoting accumulation of NK cells [112, 113]. IL-15-based activation of NK cells has been shown to lead to NASH in the murine setting [114], but interestingly, NK cells have also been shown to prevent liver fibrosis [50, 115]. This is achieved both through NK cell influence on macrophage polarisation in the liver in an NKp46-dependent manner and via killing of HSCs by NK cells in an NKG2D-dependent manner [50, 115]. In humans, NKp46-based protection against fibrosis has also been described, though in the setting of HCV [116]. Increased expression of the activating receptor NKG2D by circulating NK cells has been observed in NASH patients [117], and it is possible that this results in increased HSC killing as seen in the mouse model. Diedrich et al observed a negative correlation between fibrosis and NK cell frequency in the liver, indicating an involvement of NK cells in preventing fibrosis [118]. Interestingly, lower NKG2D expression was observed in circulating NK cells of NAFLD patients compared to healthy individuals, and a similar trend was seen in NK cells within the liver [118]. The downregulation of NKG2D in the context of NAFLD may give rise to a more pro-fibrotic NK cell population, thus contributing to worsening liver disease and fibrosis. It is possible that changes such as this are mediated by metabolic changes. Indeed, the function of NK cells has been shown to be reduced in obese patients due to excessive lipid uptake [119]. NK cells are also known to produce IL-22 following activation [58]. In mice, IL-22 has been shown to have an anti-fibrotic effect [120]; however, IL-22 has also been demonstrated to have a pro-fibrogenic role in a chronic HBV model [121], further confusing matters. Altogether, these data indicate that NK cells have a complex role in the pathogenesis of NAFLD. While NK cells appear to have a role in preventing fibrosis, changes in NKG2D-expression by NK cells accompany increasing fibrosis. Furthermore, pro-inflammatory NK cell signalling may promote NAFLD progression to NASH, and the role of IL-22 in fibrosis is still not fully understood.

## NK cell based/targeted therapies for liver disease

As NK cells play an important role in liver disease, they also have the potential to serve as a therapeutic target for these diseases. Modulation of existing NK cells can be achieved through cytokine stimulation or antibody treatment targeting either inhibiting or activating NK cell receptors. Indeed, NK cells respond to a wide array of cytokines including type I IFNs, IFNγ, IL-2, IL-12, IL-15 and IL-18. NK cells from IFNα-treated HCV patients have been shown to have increased killing of HSCs in vitro, which has been linked to a reduction in liver fibrosis [49]. IFNα treatment was shown in early studies to reduce fibrosis in HCV patients [122, 123] though more recent data indicates that it provides no benefit [124]. Of note, treatment of HCV with direct DAAs shows long-term decrease in liver fibrosis, indicating that viral clearance is sufficient for improvement [125]. Although DAAs now offer effective treatment against HCV, the same is not true for HBV. Treatment with a TLR8 agonist can indirectly activate NK cells via dendritic cell-produced cytokines, and this may prove to be an effective way to combat HBV [126].

**Fig. 5:**
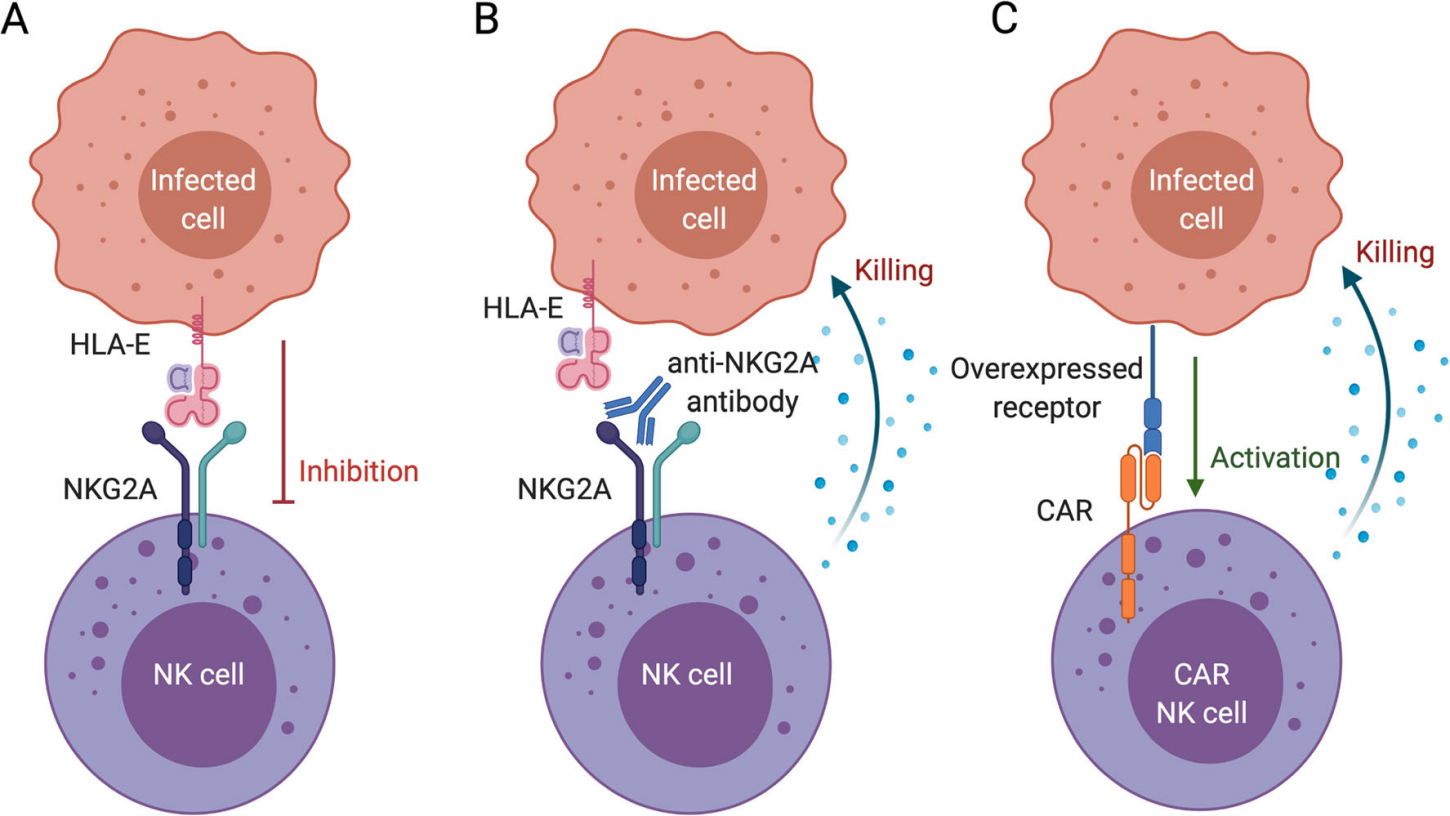
NK cell targeted therapies. A virally infected cell **A** inhibiting an NK cell via signalling through NKG2A or **B** being killed due to blockade of inhibition with anti-NKG2A monoclonal antibody. **C** A CAR NK cell recognising an overexpressed surface protein on an infected cell via an engineered CAR receptor causing activation and subsequent killing. *Created with* BioRender.com

Checkpoint blockade has transformed the treatment of melanoma by reversing exhaustion and restoring effective anti-cancer activity of T cells. As with T cells, NK cells also express inhibitory receptors such as KIRs, NKG2A and TIGIT on their cell surface to quell unwanted activation. NK cells expressing inhibitory NKG2A have been shown to be increased in chronic HBV infection [127]. Interestingly, ex vivo blockade of NKG2A increased NK cell cytotoxicity, and blockade in a mouse HBV model improved viral clearance [127]. NKG2A blockade in a mouse HCV model was also shown to improve NK cell cytotoxicity [88]. These results indicate that checkpoint blockade of NK cells may be a viable anti-viral treatment in chronic liver infection (Fig. 5). Blockade of TIGIT [128] and TIM3 [129], inhibitory molecules expressed by NK cells, is known to improve NK cell cytotoxicity against tumours. These molecules are also highly expressed on NK cells in HCV patients with advanced liver fibrosis, indicating that blockade, in this instance, may restore NK cell functionality and protection [130].

Genetically modified NK cells with chimeric antigen receptors (CAR NK cells) targeting HCC are currently being tested in multiple clinical trials, as are bi- and tri-specific antibodies designed to activate NK cells at the same time as targeting them to tumour cells [131, 132]. CARs are receptors with a specific, often antibody-derived, binding region with downstream signalling domains designed to enhance activation in the context of a specific target ligand [133] (Fig. 5). CAR NK cells and bi- and tri-specific antibodies all rely on the overexpression of specific ligands on cancerous cells. Whether these technologies can be re-purposed in the context of viral infections or liver fibrosis is yet to be seen and would rely on specific surface expression of otherwise rare ligands on target cells.

## Conclusion

NK cells are present in high abundance in the liver and thus have a large impact on the immune environment in the organ. They provide potent pro-inflammatory and cytotoxic defence against viral challenge, but dysregulation of the NK-cell response can lead to chronic inflammation and disease. Despite the importance of NK cells in the liver, much is still unknown about the exact mechanisms by which they contribute to different liver diseases, and more study is needed to understand under which circumstances NK cell subpopulations can promote or reduce liver inflammation. Discovery of distinct populations of NK cells within the liver furthermore suggests a heterogeneity in their roles. Better understanding of the specific function of different lrNK cells and cNK cells present in the liver may help to elucidate the importance of these subsets in different disease settings. Indeed, the more is known about the role of NK cells in liver disease, the more opportunity there is for targeted manipulation of these cells to ultimately improve patient outcomes.

## Data Availability

N/A.

## Code availability

N/A.
